# Qualitative study on physicians’ acceptance of a clinical decision support system for anemia management in patients receiving hemodialysis

**DOI:** 10.1186/s12913-025-13780-9

**Published:** 2025-12-29

**Authors:** Ju-Yeh Yang, Shou-Hsia Cheng, Raymond N. Kuo

**Affiliations:** 1 Division of Nephrology, Department of Internal Medicine, Far Eastern Memorial Hospital, New Taipei City, Taiwan; 2 Department of Applied Cosmetology, Lee-Ming Institute of Technology, New Taipei City, Taiwan; 3 Institute of Health Policy and Management, College of Public Health, National Taiwan University, 632R, No.17, Syujhou Rd., Taipei City, 100 Taiwan

**Keywords:** Acceptance, Anemia, Clinical decision support system, Hemodialysis, Qualitative study

## Abstract

**Background:**

Clinical decision support systems (CDSS) for renal anemia among hemodialysis patients are gaining clinical attention. However, wide adoption remains challenging, and critical factors concerning physician acceptance remain elusive. This qualitative study conducted in-depth interviews with nephrologists to determine the relevant factors influencing physician acceptance.

**Methods:**

Seventeen nephrologists in the hemodialysis center at Far Eastern Memorial Hospital participated in semi-structured, in-depth interviews to explore their views and concerns regarding CDSS. The qualitative study was performed under the well-established framework of the unified theory of acceptance and use of technology (UTAUT).

**Results:**

The majority of participating physicians (14 of the 17) believed that a CDSS could streamline their workflow and save time. All participants agreed that robust clinical evidence of CDSS efficacy was critical to enhance CDSS acceptance. In addition, physicians noted that CDSS provides educational value, promotes patient safety and quality of care, and aligns with future technological trends. Despite these benefits, physicians raised concerns regarding dependence, distrust, and potential interference, which related to the physician professionalism. The UTAUT model should be extended with additional construct of autonomy in recognition of physician professionalism.

**Conclusion:**

This qualitative study revealed that nephrologists expressed positive views on the CDSS for anemia management. They acknowledged that the CDSS could enhance their work efficiency, improve patient safety, enhance care quality, and provide educational value. To optimize the acceptance of the CDSS under the UTAUT-Autonomy framework, the design and implementation of the CDSS should also address three additional key factors: over-dependence, distrust, and workflow interruption.

**Supplementary Information:**

The online version contains supplementary material available at 10.1186/s12913-025-13780-9.

## Introduction

A clinical decision support system (CDSS) refers to software designed to assist clinical decision-making. Although a CDSS may provide clinical benefits, its clinical implementation elicits concerns such as alert fatigue, implementation costs, and workflow interference [1, 2]. According to a meta-analysis, CDSS have relatively low impact on changes in clinical healthcare behavior, with a 5.8% effect size (95% confidence interval: 4%–7.6%) [3]. Even with the aid of CDSS, more than 50% of patients do not receive the recommended treatments. Previous findings suggested that clinical healthcare personnel’s perceptions and acceptance of the effectiveness of CDSS may be critical in determining whether CDSS can substantially alter healthcare professionals’ care behaviors [4–8].

Renal anemia is an important issue in the management of patients with end-stage kidney disease, accounting for 53.4%–90% of patients [9–11]. Owing to significant variability in individual responses to anemia therapeutics, erythropoiesis-stimulating agents (ESAs), healthcare providers must conduct frequent assessments of treatment responses and dose adjustments. Consequently, numerous CDSS have been developed to assist clinical professionals in achieving stable control of anemia, reducing ESA usage, and alleviating the workload associated with clinical decision-making [12–20]. With the advancements of artificial intelligence (AI), AI-based CDSS incorporating deep learning or generative AI algorithms evolved from traditional rule-based CDSS [21]. A previous study demonstrated that physician adherence to the anemia-management CDSS is a critical mediating factor for improving anemia control [22]. However, no previous study explored nephrologists’ acceptance of CDSS in anemia management.

Consequently, this study conducted in-depth qualitative interviews to explore nephrologists’ opinions and concerns under the well-established framework of the unified theory of acceptance and use of technology (UTAUT) [23]. The UTAUT model is a framework for understanding novel technology adoption and acceptance, offering a structured approach for evaluating user perceptions and behaviors in the context of technology adoption, such as the CDSS. By examining these core constructs and addressing specific concerns, this study provides unique insights into how nephrologists perceive and interact with the CDSS in daily practice.

## Methods

### Study setting

Far Eastern Memorial Hospital (FEMH) is a medical center in Taiwan with approximately 1,400 inpatient beds, including a 110-bed hemodialysis (HD) unit. The HD unit conducts approximately 7,000 hemodialysis sessions per month. Since 2019, the HD center at FEMH has implemented an “Individualized Anemia Management Algorithm (IAMA)” CDSS [22]. This rule-based CDSS recommends individualized ESA and iron dosages based on patients’ hemoglobin and iron level changes during the previous two weeks. The IAMA CDSS automatically provides dosage recommendations, which saves time for physicians to manually review past data or perform calculations. When a prescription change is necessary, the CDSS prompts the physician with the recommended ESA and iron dosages.

### Participants

All nephrologists at FEMH, including twelve senior and six junior nephrologists, participated in this study. Demographics of participating nephrologists were collected, including age, gender, working experience, seniority, and managerial roles. Dr. JYY, a senior nephrology specialist and lead investigator of this study, conducted one-on-one interviews with all other seventeen nephrologists from July 2022 to September 2022. Interview was conducted in-person among 12 interviewees and virtually among 5 interviewees owing to COVID-19 pandemic in Taiwan. All interviewees agreed and provided informed consent before the interview, and all interviewees completed the predefined questions. The interviewees covered the entire spectra of physician experience and administrative experience in FEMH.

### Interview outline

This study employed the UTAUT model [23] as the framework and developed semi-structured in-depth interviews (Supplementary 1). The interview structure focused on the four core constructs of UTAUT: performance expectancy, effort expectancy, social influence, and facilitating conditions. Additionally, concerns regarding the CDSS were explicitly discussed. The interview outline, including sample questions, is presented in Supplemental Table 1. The structure and themes of the qualitative study had been reviewed and validated by an expert panel, which consisted of a professor whose expertise is in qualitative research. Other panelists include experts in health policy and health information systems.

All interview processes were conducted and coded by the same investigator, the lead author Dr. JYY. Although it might limit the formal assessment of inter-rater reliability, the investigator ensured consistency in iterative review and reflexive documentation throughout the analysis process. Interviews continued until no new related information emerged, and information saturation was reached [24]. In addition, at the end of each interview subsection and the end of the interview, the investigator inquired with open questions to each interviewee, “Do you have any additional ideas to share?” This ensured that the interviewees expressed their full opinions and that “information saturation” was achieved.

### Data analysis

This study employed a deductive qualitative analysis approach. All interview Q&A data were carefully read and reviewed. The coding framework was developed deductively, grounded in the four primary constructs of the Unified Theory of Acceptance and Use of Technology (UTAUT). This framework guided the initial categorization and interpretation of the interview data.

First, concepts were extracted from interviewees’ opinions. Second, extracted concepts were categorized into the main dimensions of UTAUT. If the concepts could not be assigned to UTAUT dimensions, the concepts were separately defined as additional dimensions. After repeated inspection and comparison of each concept, the concepts were reassigned into two main spectra: positive-tendency group and negative-tendency group. Each group’s concepts were re-clustered according to similarity of concepts into major categories and sub-categories. The re-clustering processes were repeated until no new categories or sub-categories emerged, thereby achieving “thematic saturation” [24]. Thematic saturation was reached when no new themes emerged in the final interviews. While data saturation was often reached after initial 12 interviews as the basis for sample size calculation in qualitative studies [25], this study observed that after approximately 15 of the total 17 interviews, no additional themes were identified, suggesting that thematic saturation had been reached. This aligns with the guidance provided by Saunders et al. [24].

NVivo Version 1.7.1 was used for data organization and analysis. The study protocol was reviewed and approved by the IEC of FEMH. The funding body for this study played no role in the design of the study or the collection, analysis, or interpretation of the data.

## Results

### Overview

The duration of each interview was approximately 30 min. They were recorded in audio format and transcribed verbatim. The interview process began with open-ended questions to explore the interviewees’ feelings and thoughts. Subsequently, the questionnaires were supplemented with guided questions to obtain specific information [23].

Seventeen physicians were interviewed. More than half were 30–39 years old (*n* = 10), followed by those aged 40–49 years (*n* = 4), and the remainder were ≥ 50 years (*n* = 3). Regarding sex distribution, the participants comprised nine males and eight females. Regarding work experience at the FEMH, four physicians had < 5 years, five had 6–10 years, five had 11–15 years, and four had > 16 years of experience. Approximately one-third of the interviewees were resident physicians (*n* = 6), and the remaining two-thirds were attending physicians (*n* = 11), with five of them serving concurrently managerial roles.

All interviewees acknowledged that the CDSS was helpful for their work. The majority of participating physicians (*n* = 14) agreed that the CDSS could streamline their workflow and save time. Additionally, all interviewees agreed that the CDSS had a clear and user-friendly interface.

### Analysis based on the UTAUT framework (Table 1)

**Table 1:** Dimensions and statements associated with physician acceptance of the clinical decision support system based on the unified theory of acceptance and use of technology (UTAUT) framework. IAMA: individualized anemia management algorithm

Dimensions/Statements		Number of interviewees concurring with the statement. (*n* = 17)
Performance expectancy	IAMA is helpful for work	17
	IAMA accelerates tasks	14
	IAMA increases productivity	15
	IAMA contributes to income or promotion	4
Effort expectancy	IAMA interface is clear	17
	IAMA is easy to understand	17
	IAMA is simple to use	17
	IAMA is easy to learn	17
Social influence	People around support the use of IAMA	14
	Significant others support the use of IAMA	13
	Supervisors support the use of IAMA	14
	Hospital supports the use of IAMA	15
Facilitating conditions	Necessary resources	15
	Sufficient knowledge	17
	System compatibility	16
	Assistance for troubleshooting	16
Attitude	Agree to use IAMA	16
	Appreciate having IAMA	17
Motivation	Keep using IAMA	17
	Use IAMA whenever possible	16
	Anticipate frequent use of IAMA	16

In the performance expectancy domain of the UTAUT framework, all interviewees agreed that the CDSS is helpful for their work. Furthermore, 14 participating physicians believed that the CDSS accelerated their tasks or saved decision-making time, and 15 believed that it increased productivity. However, only four physicians believed that it significantly contributed to increasing their income or advancing their careers. Regarding the effort expectancy domain, all respondents agreed that the CDSS interface was clear, easy to understand, simple to familiarize with, and easy to learn. Regarding the social influence domain, the majority of participating physicians reported that individuals around them (*n* = 14), significant others (*n* = 13), supervisors (*n* = 14), and hospital authorities (*n* = 15) supported the use of CDSS. Regarding the facilitating conditions domain, key facilitating factors included access to necessary resources for using the CDSS (*n* = 15), sufficient knowledge (*n* = 17), system compatibility (*n* = 16), and availability of assistance for troubleshooting (*n* = 16), with the majority of participating physicians providing affirmative responses. Nearly all participating physicians held a positive attitude toward the CDSS. Particularly, 16 of the 17 interviewees agreed that using the CDSS is a good idea, and all of them appreciated having the CDSS to assist with their work. Moreover, the participating physicians demonstrated high motivation to continue using the CDSS; all interviewees intended to keep using it, 16 of them actively tried to use the system whenever possible, and anticipated frequent use of the CDSS.

### Physicians’ perceptions of the CDSS

Despite the positive attitudes, the interviewees estimated that only 5–9 out of 10 recommendations made by the CDSS were adopted. When asked how to increase CDSS acceptance, all participating physicians agreed that robust clinical evidence was the most critical determinant. Moreover, peer recognition and directives from superiors also influenced their CDSS acceptance.

Physicians’ perceptions of the CDSS can be summarized and divided into two main categories: those who acknowledge the benefits of the CDSS and those who are concerned about it (Table 2).

**Table 2:** Summary of physicians’ perceptions of the clinical decision support system

Main category	Category	Concepts
Acknowledgment	Work Efficiency	CDSS extracts the necessary information for decision-making and saves time for physicians. If appeared reasonable, physicians would adopt CDSS recommendations without reevaluating patient’s data.
	Education and Learning	CDSS recommendations serve as a valuable teaching resource. Physicians can have an opportunity for reflection and self-improvement.
	Patient Safety and Care Quality	CDSS prevents omissions, enhancing patient safety. CDSS fosters consensus among different physicians.
	Future Trend	AI-assisted CDSS might surpass human judgment. Still room to develop other CDSS, such as phosphate control therapy and ultrafiltration volume settings.
Concern	Over-Dependency	Physicians may not have the opportunity to cultivate their professional judgment and experience. Physicians may believe that they do not need to act if the CDSS does not indicate a recommendation.
	Distrust	The parameters and duration evaluated by algorithms are insufficient. The algorithms cannot handle untrained scenarios. Still need data on treatment-related outcomes. Acceptable consistency between CDSS and physician judgement and convincing data on savings in time and cost.
	Threats to Professional Autonomy	Physicians are still responsible for prescriptions. Under normal use, CDSS would not affect physicians’ judgment.
	Interference by alert fatigue and extra workload	The majority of interviewees experienced alert fatigue and admitted the possibility of ignoring CDSS recommendations. Physicians do not want the CDSS to increase their workload.

#### Acknowledgment of the CDSS: work efficiency

The CDSS streamlines clinical decision-making by extracting the necessary information for decision-making and reducing the time that physicians spend on data retrieval and assessment. This expedites clinical judgment, aids decision-making, and serves as a valuable reference, particularly in challenging situations. Some physicians admitted that they occasionally forego re-evaluations and directly adopt CDSS recommendations, particularly when they are busy. The interviewees stated:

When we do not have much time to think, it is crucial to at least have a rough direction in mind. (resident physician)

I think it checked the previous changes and got a recommended dosage, so I do not have to figure it out myself. (attending physician)

If I feel like my judgment aligns closely with the recommended dosage, I just go with that and skip the hassle of figuring it out or calculating it myself. (resident physician)

#### Acknowledgment of the CDSS: education and learning

Regarding colleagues with limited clinical experience, CDSS recommendations serve as a valuable teaching resource. Even when the CDSS suggestions differ from their own, physicians can use these differences as reflection and self-improvement opportunities. Among the participants, 11 physicians acknowledged that the CDSS recommendations could stimulate critical thinking, contribute to professional growth, and provide opportunities to review their thought processes. The interviewees shared:

In fact, it makes me reflect on my judgment. Overriding the system pushes me to think deeper. It’s somewhat stimulating—we can’t claim to know everything ourselves, right? (attending physician)

It’s like the CDSS approaches things differently, adding another layer of logic. Our thinking can get rigid—this offers stimulation from an alternative source, leading to different outcomes. (resident physician)

#### Acknowledgment of the CDSS: patient safety and quality of care

The CDSS feature “recommendations” was designed to prevent omissions and enhance patient safety. The interviewees stated:

Clicking on it and seeing red text signals that there might be an issue, reminds me. Otherwise, it is easy to overlook things at times. (attending physician)

It at least has an alert function—reminding me there’s something to adjust, prompting me to assess and reevaluate the patient’s current condition. (attending physician)

Four physicians mentioned that the CDSS fostered consensus among different personnel and that consistency in the treatment approaches of all nephrologists in the same dialysis unit resulted in improved patient care. One interviewee mentioned:

It’s not just a suggestion; it helps establish consensus or routine, allowing everyone to take a more consistent approach to prescribing ESAs. (attending physician)

#### Acknowledgment of the CDSS: future trends

The participating physicians uniformly agreed that AI-based CDSS is an ongoing trend that may surpass human judgment. The interviewees generally supported the continued development of other CDSS tools for dialysis care, such as phosphate control therapy and ultrafiltration volume settings. One physician stated:

This system aligns with evolving trends—humanity gradually integrating technology. It’s like replacing part of the human brain with AI. (attending physician)

#### Concerns about the CDSS

The primary concern among the participating physicians regarding the CDSS was a lack of trust in the system. They suggested that the CDSS algorithms had several shortcomings and lacked solid evidence. Sixteen physicians mentioned the current limitations of rule-based CDSS algorithms, such as insufficient individualization owing to limited parameters (10 physicians), inability to handle unforeseen circumstances (10 physicians), insufficient reference duration (7 physicians), and insufficient sensitivity (7 physicians). Some participants also mentioned the lack of a theoretical basis for CDSS recommendations and the absence of consensus regarding treatment goals.

All participating physicians agreed that, whether a rule-based or data-driven CDSS is used, solid evidence is needed to demonstrate its effectiveness before healthcare providers can confidently adopt its recommendations. The types of empirical evidence mentioned included an array of anemia treatment-related indicators, such as average hemoglobin values, target hemoglobin maintenance rates, stability, the proportion of extremely abnormal hemoglobin values, and consistency between the CDSS and physician judgment. Moreover, evidence of time or cost savings by the CDSS was mentioned as well.

The second concern was the fear that the CDSS may lead to overdependence. Twelve participating physicians mentioned the risk of dependency, particularly for less experienced physicians, who may not yet harness professional judgment skills and experience. However, the interviewees believed that this dependency could be avoided through education and self-imposed requirements. Some physicians also believed that they might not proactively adjust medications if the CDSS did not provide recommendations. Several interviewees expressed such concerns:

It will definitely lead to dependence, but I think it comes down to the individual competence and behavior of the physicians. (attending physician)

There’s a concern doctors might rely too much. Once they trust the system blindly, they stop understanding why. That’s the key issue—we need to strengthen this in medical education. (attending physician)

For younger physicians, relying too much on the program risks weakening their own judgment. If it’s taken away, they might struggle to adjust medications—a concern worth addressing. (attending physician)

Our qualitative interviews suggested differences in attitudes on CDSSs based on physicians’ level of seniority. Overall, senior attending physicians (aged 40 and above) viewed CDSS positively, emphasizing its utility in enhancing clinical efficiency, reducing oversight, and promoting consistency and standardization in prescribing practices. Nevertheless, several senior physicians explicitly cautioned that junior physicians without sufficient clinical experience might rely too heavily on system suggestions, thereby neglecting inter-patient clinical variability and potentially making inappropriate treatment decisions. For example, one attending physician over 50 noted, “This system does not account for individual variations. It applies a uniform logic, and if an inexperienced physician follows it blindly, there could be problems.”

By contrast, residents and junior attending physicians aged 30–39 generally acknowledged the value of CDSS recommendations but primarily used them as reference points, deferring to their own clinical assessments. Some younger physicians indicated that due to the lack of transparency in the CDSS’s logic or its inability to account for inpatient data dynamics, they often chose to disregard or only loosely follow the recommendations. Others noted that as they became more familiar with the CDSSs and accumulated clinical experience, they relied less on CDSS and instead applied their own judgment in adjusting dosages.

The physicians did not believe that the CDSS threatened their professional status or autonomy. They believed that medical practice requires a high level of expertise and that it would only be affected if there was a lack of inherent professionalism. No physician believed that the CDSS would affect their professional judgment under normal use. The interviewees stated:

The system itself would not pose a threat to me because it does not have any mandatory elements. (attending physician)

We are nephrologists, and we are familiar with things such as dialysis. I do not think it will impact our professional judgment. (attending physician)

Although some physicians admitted that they occasionally forwent re-evaluation and directly adopted CDSS recommendations, only five physicians believed, under specific circumstances and with strong evidence, that the CDSS could directly replace physician judgment. The remaining 12 physicians believed that, regardless of the strength of the CDSS evidence, it must not generate prescription orders directly and that physicians, not the CDSS, still held accountability for prescriptions. The physicians stated:

Assuming you’re mandated to accept it, it affects autonomy. It’s no longer assistance—it’s coercion, a system placing itself above the physician’s judgment. (attending physician)

In the end, it still needs human authorization. Whether AI and machines can replace humans might be a possibility in the future. At the current stage, I find it unlikely. (attending physician)

Regarding concerns about alert fatigue caused by the CDSS, the majority of interviewees echoed similar experiences and admitted the possibility of ignoring CDSS recommendations. However, they believed that the current CDSS design for anemia treatment would not interfere with or disrupt their work. The majority of interviewees considered the current level of prompting to be adequate. Additionally, some interviewees did not oppose increasing the intensity of recommendations, but did not want the CDSS to increase the workload. The physicians shared their experience:

The reason clinical support systems feel like a hindrance is they constantly interrupt your workflow, and if you don’t follow their instructions, you get stuck. (resident physician)

It is like our outpatient system has many pop-ups, various types of them to click through. Having a window popping up that you have to dismiss can be disruptive. (attending physician)

## Discussion

According to our knowledge, our study is the first qualitative study investigating relevant factors of physicians’ acceptance of a CDSS in anemia management, based and extended from the technology acceptance and adoption model (UTAUT).

The interviewed nephrologists acknowledged that the CDSS could alleviate their workload. However, their acceptance was contingent on the evidence of effectiveness. The majority of participating physicians did not believe that a CDSS could directly replace physicians’ prescriptions, nor did they consider the CDSS a threat to their autonomy or professional status. The primary concern was the potential for overdependency on the CDSS, particularly among less experienced physicians. The results underscore a structural divergence in how senior and junior physicians perceive the role and function of CDSSs. Senior physicians focus on the system’s potential for standardizing prescriptions and enhancing process consistency, and many support automated implementation where evidence is robust. In contrast, junior physicians value the supplementary role of CDSS in supporting clinical judgment and emphasize retaining final discretion to safeguard patient safety. The possibly differential impact of overdependence to either senior or junior physicians deserves further investigation in a prospective study.

Our study’s findings indicated that physicians held both positive and negative perspectives concerning the CDSS. For example, physicians simultaneously acknowledge the educational value of the CDSS while expressing concerns about its impact on the development of their professional judgment. They hoped that the CDSS would assist in improving the quality of care, but also feared that its mandatory recommendations would increase interference. They expected that AI-based CDSS could outperform physicians’ judgment, but simultaneously believed that their medical professional status and autonomy would not be threatened. They hoped to mitigate their concerns through education and self-discipline.

This qualitative study was conduct after three years of implementation of IAMA, instead of early adoption of IAMA. This chronological timepoint and familiarity of the IAMA by physicians after three years may also contribute to favorable perceptions among physicians, compared to early adoption phases. Though beyond the scope of this qualitative study, chronological variations in the physician acceptance might need to be considered when interpreting our study results. In the early implementation of CDSSs, physicians might have lower acceptance owing to less familiarity, inertia to change, and under-appreciation of the incremental benefits of the new CDSSs. When interpreting at late phase, such as in our study, physicians may already have better familiarity, less inertia, and more appreciation of the incremental benefits, which contribute favorable acceptance of the CDSSs.

This study’s results suggested that the factors in the UTAUT model were insufficient to explain the low acceptance rate of physicians toward IAMA recommendations. Despite high levels of agreement among respondents across all dimensions, including perceived usefulness, perceived ease of use, social influence, and facilitating conditions, as well as nearly 100% motivation and behavioral intention, the actual adoption rate of IAMA recommendations by physicians remained low. Our previous quantitative analysis revealed that the estimated consistency rate between physician prescriptions and IAMA recommendations was 78.6% [22]. This finding indicated that physicians’ acceptance of the CDSS could not be fully explained by extant technology acceptance models.

Although UTAUT models have been utilized to explain the acceptance of information technologies in clinical applications, the explicability was incomplete [23]. Three prior qualitative studies on adoption of technologies in medical applications had proposed extended UTAUT models by discovering additional constructs beyond the four traditional UTAUT constructs. For example, Alaiad et al. investigated the adoption of home healthcare robots and identified additional constructs of trust, privacy concerns, and ethical concerns beyond the UTAUT model [26]. Hoque et al. studied the adoption of mobile health among elderly subjects, and suggested additional constructs of technical anxiety and resistance to change [27]. Furthermore, Mailet et al. explored the acceptance and actual use of electronic patient records by nurses, and found that compatibility of the electronic patient records and computer self-efficacy were two additional meaningful constructs beyond the UTAUT model [28]. Liu et al. introduced the construct of “motivational control” in addition to the UTAUT model. This construct is like the concept of “autonomy,” which is unique to humans [29]. Given that autonomy is a fundamental characteristic of professional roles, Liu et al. suggested that the CDSS might be perceived as a threat to physicians’ professional autonomy, potentially negatively affecting their acceptance [29]. Previous studies indicated that healthcare professionals are concerned that the CDSS could threaten their professional autonomy, which contributes to their reluctance to adopt CDSS recommendations [30].

This qualitative analysis revealed several perspectives that are not fully captured by the current four UTAUT dimensions, including: (I) Concerns about over-dependence on the CDSS, particularly among less experienced physicians, (II) Distrust of CDSS recommendations, owing to perceived limitations or insufficient transparency, and (III) Workflow interruptions caused by system alerts or CDSS design. These factors reflect physicians’ need to maintain professional autonomy and control in clinical judgment, which are dimensions that are central to CDSS acceptance but not explicitly addressed by the UTAUT model. This study advocates that incorporating autonomy-related constructs may enhance the explanatory power of UTAUT in interpreting physicians’ acceptance of the CDSS. Based on the findings of this study, we recommend that the UTAUT should be expanded into “UTAUTA”, adding the “Autonomy” construct to incorporate the critical factor of physician autonomy.

The majority of nephrologists in this study did not perceive the current rule-based CDSS (IAMA at FEMH) as a threat to their professional autonomy or could replace or influence physicians’ judgment.

However, in the era of AI-based CDSS, clinical professionals may find it challenging to understand the “black box” algorithms beneath the CDSS. Furthermore, the CDSS outperforming even the most seasoned physicians in some scenarios may elicit distrust and concerns [31]. In a recent qualitative interview study on physicians’ adoption of AI-based CDSS, Vijayakumar et al. identified evidence, patient safety, data availability, awareness, and collaborative functioning as key aspects in defining physician adoption [32]. The rapidly advancing generative AI and large language model (LLM) programs (ChatGPT, Gemini, Llama, Claude) continuously reshape the landscape of clinical decision-making concerning diagnostic assessment [33], therapeutic interventions [34], prognostic assessment [35], and unravelling new insights [36]. Notably, Yu et al. emphasized the importance of human value during the training data, model development, and model use [37]. Omar et al. cautioned about the potential sociodemographic biases embedded in LLM-based clinical decision-making [38]. Our study findings expanded the technology adoption model into the UTAUTA framework by adding the physician autonomy construct, which might reduce the concerns of “black box” AI-based CDSSs from physicians. Specifically, when designing and implementing AI-based CDSSs, the developing team should incorporate physicians into the designing input (“human-in-the-loop” design) and constantly fine-tune the algorithms based on physician’s comments. In the work of user interphase design and system implementation, physicians should play active roles in order to integrate the CDSSs into daily workflow and explicitly demonstrate the incremental benefits and clinical value of the CDSSs. Future studies should explore how physicians perceive the threat to their professional autonomy by AI-based CDSS and any discrepancy between perceived acceptance and actual adoption in prospective studies. For example, the prospective study could firstly record pre-implementation physician perceptions on CDSSs, secondly measure the discrepancies between the physician prescriptions and CDSS recommendations during the implementation, and lastly record the physician perceptions on CDSSs after the implementation both in the early adoption and late adoption phases.

Moreover, the interviews revealed that the complexity of medical decision-making extends beyond the logic of CDSS algorithms. Different physicians have varying treatment principles and often do not adhere to medical guidelines. Additionally, they have to consider insurance regulations, performance metrics, hospital policies, treatment costs, personal experiences, and patient preferences, which considerations may not always align with the goals or logic of the CDSS.

Our findings emphasized that physicians’ adoption of CDSS is influenced by unique professional considerations that differ from general consumers’ acceptance of daily life technologies. In the clinical context, professional autonomy and the primacy of human judgment remain critical. As emphasized in a recent review article [37], regardless of the advancement of AI in healthcare, human value remains central to medical decision-making. Therefore, successful implementation of CDSS requires not only alignment with UTAUT dimensions but also deliberate efforts to ensure that CDSS does not undermine physicians’ autonomy, especially in current era of AI-based CDSS. Future implementation strategies include enhancing transparency through explainable AI (XAI) models [39–43], integrating physician’s input during the training of AI-based CDSS (human-in-the-loop), and designing alert systems in the AI-based CDSS that support, rather than disrupt, the clinical workflow [44–46]. The implementation of AI-based CDSS should incorporate and respect the discretion of the physician, respecting the irreplaceable roles of physicians’ human care for patients, professional judgement, and ethical responsibility in clinical medicine.

A limitation of this study was that the sample only included a single medical center. However, the Far Eastern Memorial Hospital (FEMH) Hemodialysis Center is a large tertiary care facility in Taiwan, serving a high-volume patient population and staffed by a diverse group of nephrologists with varying levels of clinical experience and managerial responsibility. The sample included all eligible nephrologists at the center, encompassing a range of professional roles and years of practice.

Although the findings of qualitative studies were context-specific, the heterogeneity of participants within this institutional setting enhanced the potential transferability and generalizability of the findings, particularly to other large-scale hemodialysis units. Meanwhile, FEMH is a private tertiary referral medical center, where the top managers’ leadership and requirements to meet the hospital accreditation encourage the pursuit of excellence in CDSSs. This culture might overly stimulate physicians’ acceptance of CDSS, as compared with physicians in public hospitals or in smaller facilities with different technological cultures. In contrast, healthcare organizations with less mature technological cultures—such as limited IT infrastructure, weaker managerial support, or lower digital confidence among clinicians—may experience greater resistance to adoption. In medical institutions with the institutional culture of less emphasis on technological advancements, physicians might have less awareness or less motivated to adopt new technologies such as CDSSs. Such cultural factors of different technology adoption should be considered when making the generalization of our study results to other healthcare systems [47]. An organization’s culture—its ingrained values, norms, and practices—creates a unique environment that can either foster or hinder the adoption of new technologies [48, 49]. The implementation of CDSS is inherently influenced by the “technological culture” within healthcare systems, encompassing clinicians’ digital literacy, institutional support for innovation, and the broader sociotechnical context [8, 50]. Tailored implementation strategies, including gradual implementation, capacity building, and participatory design that involve clinicians, are crucial to ensuring the sustainable integration of CDSS. Future research should investigate how variations in technological culture influence the adoption and long-term use of CDSS.

Previous studies suggested that organizational factors such as the hospital size, level of advanced care, and degree of IT support might influence physicians’ adoption and opinion on the CDSS. Therefore, further research in diverse settings is needed to explore how contextual factors would shape CDSS acceptance across different medical institution settings [4, 6, 7].

Despite these limitations, our qualitative study with in-depth interviews provides unique value in elucidating frontline healthcare professionals’ conflicting thoughts on the CDSS. These unique findings could help optimizing the design and implementation of CDSS in the future to increase physician acceptance. In addition, generative AI has advanced rapidly, physicians’ views on the AI-based CDSS, particularly concerning credibility and perceived threats, are likely to change as they recognize the ever-growing capabilities of generative AI. Future studies should examine this transition for comparative purposes.

Our qualitative study exploring the physician acceptance of CDSS is timely in the rapidly advancement of AI in CDSS. With all the possible future research directions, we believe that a prospective study would bring the most clinical impact with pre-, during, and post-implementation of CDSS with recordings of pre-CDSS physician perception, measurements of the discrepancies between the physician prescriptions and CDSS recommendations, and post-implementation physician acceptance post both in the early adoption and late adoption phases. Such prospective study results would bring illuminating insights into the impact of physician acceptance and the clinical impact of CDSS. Furthermore, to advance the proposed UTAUT-Autonomy framework beyond conceptual development, future quantitative studies should operationalize the autonomy construct through measurable dimensions such as perceived professional discretion, decision-making authority, and freedom from external control [29]. Incorporating these variables into structural equation modeling (SEM) could enable empirical testing of their interactions with the traditional UTAUT constructs. Additionally, cross-institutional and cross-cultural validation in healthcare systems with varying technological maturity would help establish the generalizability of the UTAUT-Autonomy framework and clarify how autonomy modulates physicians’ acceptance and sustained use of CDSS.

## Conclusions

This qualitative study revealed that nephrologists expressed positive views on the CDSS for anemia management. They acknowledged that the CDSS could enhance their work efficiency, improve patient safety, enhance care quality, and provide educational value. They further recognized the CDSS as a trend worthy of further promotion. However, this study revealed that physicians expressed concerns regarding the CDSS, predominantly related to its credibility and reliance. To optimize the acceptance of the CDSS by physicians under the UTAUT-Autonomy framework, the design and implementation of the CDSS should also address three additional key factors: concerns about over-dependence, distrust of CDSS recommendations, and workflow interruptions.

## Supplementary Information

Below is the link to the electronic supplementary material.

Supplementary Material 1

## Data Availability

The datasets generated during the study are not publicly available but are available from the corresponding author upon reasonable request.

## Abbreviations

AI: Artificial intelligence
CDSS: Clinical decision support system
ESAs: Erythropoiesis-stimulating agents
FEMH: Far Eastern Memorial Hospital
HD: Hemodialysis
IAMA: Individualized Anemia Management Algorithm
UTAUT: Unified theory of acceptance and use of technology
