# Supplementary material for: Qualitative study on physicians’ acceptance of a clinical decision support system for anemia management in patients receiving hemodialysis
Source: BMC Health Serv Res. 2025 Dec 29;25:1620. doi: 10.1186/s12913-025-13780-9 (PMC12751585; doi:10.1186/s12913-025-13780-9)
Supplement: Supplementary file 1 — Supplementary Material 1 [file 12913_2025_13780_MOESM1_ESM.pdf]

Supplement 1. Outline of Interview Questions.

Outline	Open questions	Examples of Guiding Questions
Experience	● How has your experience been using IAMA?<br>● How does IAMA impact your work?	● Do you usually follow IAMA suggestions? Why or why not?<br>● Do you typically skip IAMA suggestions? Why?<br>● Do you generally refer to IAMA suggestions? Why?<br>● If you find IAMA suggestions unreasonable, what is your usual course of action?<br>● Does IAMA have an impact on your work?
Concern	● What concerns do you have about using IAMA?<br>● What factors would make you reluctant to use IAMA?	● Do you believe that IAMA suggestions would influence your professional judgment?<br>● Do you think IAMA poses a threat to your professional standing?<br>● Are you concerned about relying on IAMA in the future?<br>● Would you prefer IAMA suggestions to be implemented directly without the need for physician approval?<br>● If you could understand the algorithmic rules or logic behind IAMA, would you be more accepting of its suggestions?<br>● Do you think there should be empirical evidence for the effectiveness of IAMA?
Suggestion	● What suggestions do you have for IAMA?	● Do you agree with the way IAMA presents suggestions?<br>● Do you agree with the timing of IAMA suggestions?

		● Do you agree with the logic behind IAMA suggestions?
Factors associated with acceptance		
Performance expectancy	● "What benefits do you experience from using IAMA?	● Do you believe IAMA is helpful in your job?<br>● Does using IAMA help you complete tasks more quickly?<br>● Does using IAMA increase your productivity?<br>● If you use IAMA, do you have a greater chance of increasing income or advancing in your position?
Effort expectancy	● Does using IAMA require any effort on your part?<br>● Are there any difficulties you encounter when using IAMA?	● Is your interaction with IAMA clear and understandable?<br>● For you, is using IAMA straightforward?<br>● Do you find IAMA easy to use?<br>● Is learning how to use IAMA easy for you?
Social influence	● What impact does the people around you have on your use of IAMA?"	● Do most influential individuals in your life (superiors, colleagues, etc.) think you should use IAMA?<br>● Do most important people in your life (superiors, colleagues, etc.) believe you should use IAMA?<br>● Is the management supportive of using IAMA?<br>● In general, does the hospital support the use of IAMA?
Facilitating conditions	● What factors would encourage you to use IAMA?	● Do you have the necessary resources for using IAMA?<br>● Do you possess the required knowledge for using IAMA?<br>● Is this IAMA system incompatible with other

		systems you use?<br>● When you encounter difficulties with IAMA, is there someone dedicated to assisting you?
Attitude	● What is your opinion on IAMA?	● Do you think it's a good idea to use IAMA?<br>● Do you enjoy having IAMA assist with your work?
Intention	● What is your motivation for using IAMA?	● Do you intend to use IAMA?<br>● Do you anticipate using IAMA in the future?<br>● Do you plan to use IAMA in the future?
